# Association between clonal hematopoiesis-related gene mutations and unfavorable functional outcome in patients with large-artery atherosclerotic stroke

**DOI:** 10.1186/s40001-023-01566-w

**Published:** 2023-12-16

**Authors:** Xin Qiu, Jiaxu Weng, Yingyu Jiang, Lingyun Cui, Hongqiu Gu, Yong Jiang, Yalun Dai, Hao Li, Yongjun Wang, Zixiao Li

**Affiliations:** 1 Department of Neurology, Beijing Tiantan Hospital, Capital Medical University, No 119 South 4th Ring West Road, Fengtai District, Beijing, 100070 China; 2 China National Clinical Research Center for Neurological Diseases, Beijing, China; 3 Advanced Innovation Center for Human Brain Protection, Capital Medical University, Beijing, China; 4 Research Unit of Artificial Intelligence in Cerebrovascular Disease, Chinese Academy of Medical Sciences, Beijing, China; 5 Center for Excellence in Brain Science and Intelligence Technology, Chinese Academy of Sciences, Shanghai, China; 6 Chinese Institute for Brain Research, Beijing (CIBR), Beijing, China

**Keywords:** Clonal hematopoiesis, Stroke, Functional outcome, Interleukin-6

## Abstract

**Background:**

Clonal hematopoiesis of indeterminate potential (CHIP) is a phenomenon that characterizes individuals with somatic mutations that are related to hematologic malignancy but without hematologic abnormalities. Presence of CHIP is associated with the atherosclerotic cardiovascular disease through the activation of the interleukin 6 (IL-6) pathway; however, its role on unfavorable functional outcomes in different etiologies of ischemic stroke remains unclear. We aimed to investigate the association between CHIP-related gene mutations and unfavorable functional outcomes of ischemic stroke with different etiologies.

**Methods:**

We prospectively studied a cohort of 3396 stroke patients with identified etiologies, and identified CHIP and the presence of the *IL6R* variant (*IL6R p.Asp358Ala*) by whole-genome sequencing. The *IL6R p.Asp358Ala* coding mutation was used as a genetic inhibition for IL-6 signaling. The primary outcome was unfavorable functional outcome [(Modified Rankin Scale), mRS 2–6] at 3 months.

**Results:**

Of the 3396 patients, 110 (3.2%) were CHIP carriers and the median age was 62 years (IQR, 54.0–69.0). The CHIP increased the risk of unfavorable functional outcome among patients with hyper-inflammation status of high-sensitivity C-reactive protein (hsCRP) > median levels in patients with large-artery atherosclerosis (LAA) (OR 2.45, 95% CI 1.00–5.98, *p* = 0.049, *p*_interaction_ = 0.01). Presence of *IL6R* variant (*IL6R p.Asp358Ala*) could attenuate the risk of unfavorable functional outcome only in patients with CHIP (OR 0.30, 95%CI 0.12–0.76, *p* = 0.01, *p*_interaction_ = 0.02), and especially in LAA patients with CHIP (OR 0.1, 95%CI 0.02–0.42, *p* = 0.002; *p*_interaction_ = 0.001).

**Conclusion:**

CHIP is associated with unfavorable functional outcomes in patients with LAA stroke and hyper-inflammation. Genetic IL-6 signaling inhibition might attenuate the risk of unfavorable functional outcomes in CHIP carriers, especially in LAA stroke patients.

## Introduction

Clonal hematopoiesis of indeterminate potential (CHIP) refers to the existence of clonal expanded hematopoietic stem cells caused by somatic mutations in individuals without evidence of hematologic malignancy or other hematologic abnormalities [1]. Previous studies have shown that CHIP overly activates some specific inflammatory pathways and accelerates the development of atherosclerosis [2]. Experimental studies confirmed that mice with *TET2*-driven CHIP have larger atherosclerotic lesions in the aortic root, which might be caused by the altered transcriptional output of inflammatory chemokines such as interleukin 6 (IL-6) in macrophages [2]. Previous studies indicated that *IL6R p.Asp358Ala* [a commonly occurring variant in the IL-6 receptor (*IL-6R*) gene] could disrupt IL-6 signaling and provide a genetic proxy for tocilizumab, which reduces the risk of cardiovascular diseases in CHIP carriers [3, 4].

As a novel risk factor, CHIP is associated with a 14% increase in the odds of incident stroke, which is a life-threatening condition with complex etiologies. According to the Trial of Org 10172 in Acute Stroke Treatment (TOAST)—the most commonly used classification of ischemic stroke etiology—three identified etiologies were included: large-artery atherosclerosis (LAA), cardioembolism (CE), and small-artery occlusion (SAO) [5]. Arends et al. [6] have demonstrated that CHIP was associated with LAA stroke, indicating that CHIP might play a specific role in the pathogenesis of this subtype of ischemic stroke. The role of CHIP in the functional outcomes of stroke with different etiologies remains unknown. Hence, this study aimed to investigate the associations and underlying mechanisms between CHIP and the unfavorable functional outcome of stroke in different etiologies.

## Methods

### Study cohort

**Fig. 1:**
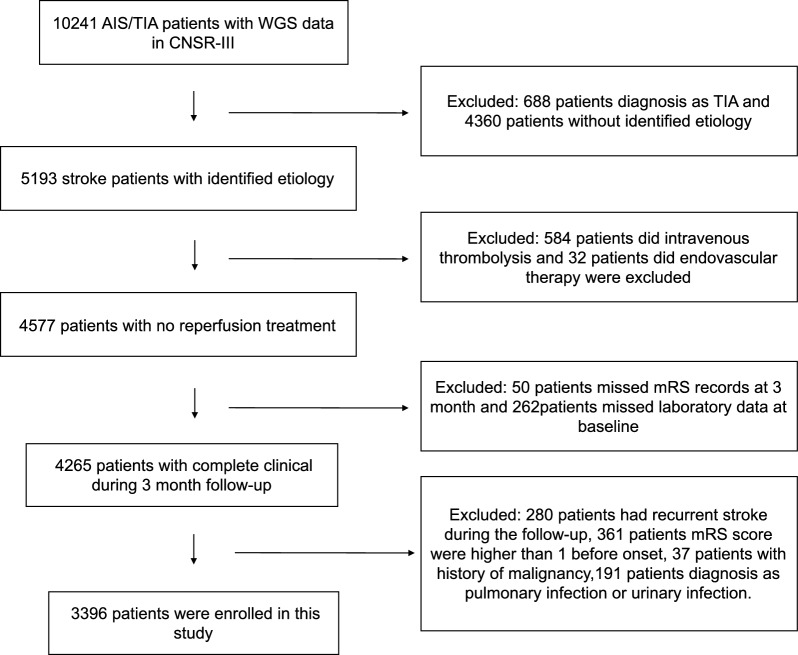
Flowchart of patients’ enrollment

We selected all patients from a prospective cohort of patients who presented to 201 hospitals with acute ischemic stroke (AIS) or transient ischemic attack (TIA) within 7 days from the onset of symptoms to admission from different regions of the country in China between August 2015 and March 2018 [7]. The final diagnosis of acute ischemic stroke was based on the World Health Organization criteria and confirmed by imaging results. Classification of etiology was performed strictly according to the TOAST criteria [5]. After excluding patients with TIA, we included patients classified with an identified etiology of stroke (including LAA, CE and SAO) and without a history of malignancy in this study. To reduce the confounding factors of functional outcome, we also excluded patients with recurrence during the 3-month follow-up, those with disabilities [modified Rankin Scale score (mRS) > 1] before the onset of symptoms, patients with reperfusion treatments including intravenous thrombolysis and endovascular therapy, and those with infection. A detailed flowchart of patient enrollment is shown in Fig. 1. The ethics committee of Beijing Tiantan Hospital and all participating centers reviewed and approved the protocol. All participants or legally authorized representatives signed informed consent forms before study recruitment into this study.

Clinical information, imaging, and laboratory data were prospectively collected. We collected blood samples from all patients as soon as they arrived at the hospital. High-sensitivity C-reactive protein (hsCRP) levels were measured at Beijing Tiantan Hospital. At 3 months after initial admission, the patients underwent follow-up including a face-to-face interview. The primary outcome in our study was 3-month unfavorable functional outcome, defined as an mRS of 2–6 [mRS; range 0 (no symptoms) to 6 (death)] [8]. We used hsCRP, a marker of inflammation, to evaluate the inflammatory status of individuals [9]. HsCRP > median levels (calculated in different subgroups) was defined as hyper-inflammation.

### Whole-genome sequencing (WGS)

Total DNA was isolated from the peripheral white blood cells (WBCs) of enrolled patients using the Magnetic Blood Genomic DNA Kit (DP329, TIANGEN Biotech Co Ltd, Beijing, China) and the KingFisher Flex system (Thermo Scientific Co, Massachusetts, USA). The qualified DNA samples were sequenced by the BGISEQ-500 platform (BGI Genomics, Shenzhen, China). All raw sequence data were stored in the FASTQ format. The average sequencing depth of WGS for each subject was greater than 30×. Raw data were processed using the following steps: both paired reads were removed if either of the reads (1) contained a sequencing adapter; (2) had a low-quality base ratio above 50% (base quality ≤ 12); (3) or contained an unknown base (‘*N*’ base) ratio over 10%. Subsequently, we used fastp (V.0.20.0) to filter out low-quality reads and bases, and conducted downstream bioinformatic analyses using these data. Downstream bioinformatics analyses were conducted on the curated dataset: reads were aligned to the hg38 human reference genome sequence that downloaded from the Genome Analysis Toolkit (GATK) bundle (ftp://www.gsapu-bftp-anonymous @ ftp://www.broadinstitute.org/bundle/hg38/Homo_sapiens_assembly38.fasta.gz). More details on the WGS methods used in our CNSR-III cohort have been reported previously [10].

### Somatic mutation identifying

The identification of somatic variants was performed using GATK MuTect2 software. A variant allele fraction (VAF) of 0.05, minimum variant read counts of 3, and coverage of 10, were used to call potential somatic variants with MuTect2. We obtained a public panel of reference genomes using GATK MuTect2 software (gs://gatk-best-practices/somatic-hg38/1000g_pon. hg38.vcf.gz). Mutations in the regions that we used for subsequent analysis were previously reported in the literature and/or in the Catalog of Somatic Mutations in Cancer (COSMIC, http://cancer.sanger.ac.uk/cancergenome/projects/cosmic/). As previously reported, we conducted an exact binomial test to eliminate possible germline variations [2, 11]. Variants with VAF significantly different from the expected distribution were considered as somatic mutations, and more details were previously published [12]. Pathogenic variants that were known to be related to the myeloid malignancies in the exonic regions of 74 genes were defined as CHIP [2].

### IL-6 inhibition genetic proxy

As previously reported [4], we used the IL-6 receptor disruptive coding mutation, *IL6R p.Asp358Ala* (rs2228145), as a genetic proxy for IL-6 pathway inhibition. This allowed us to evaluate the potential efficacy of targeting IL-6 therapy to reduce the risk of an unfavorable functional outcome in CHIP carriers.

### Statistical analysis

Continuous variables were presented as means with standard deviation (SD) or medians [interquartile range (IQR)]. Categorical variables were presented as numbers (percentages). Student’s *t*-test was used for continuous variables, while the Chi-squared test was used for comparison of response rate difference. We evaluated the correlation between the mRS and CHIP using odds ratios (ORs) and calculated it using logistic regression. The logistic regression model was adjusted using stabilized inverse probability weights (IPTW), used to reduce the influence of very small estimated probabilities from the propensity score model. We assessed whether the CHIP effect differed in different inflammation statuses or the treatment effect of genetic IL-6R inhibitors differed in different CHIP-carrier statuses by testing the interaction effect with the use of logistic regression models adjusted by stabilized IPTW. Statistical significance was set at a two-sided *p*-value < 0.05. We used the SAS software, V.9.4 (SAS Institute Inc) software for all statistical analyses.

## Results

### Characteristics of the participants

There were 5193 patients with stroke of identified etiology in The Third China National Stroke Registry (CNSR-III). After excluding 616 patients who underwent reperfusion treatments (584 intravenous thrombolysis and 32 endovascular therapy), 43 patients with missing outcome data at 3 months, 242 patients with missing clinical data, 242 patients with recurrent stroke during a 3-month follow-up, 317 patients with mRS > 1 before the onset of symptoms, 50 patients with a history of malignancy, and 287 patients with concomitant infection, and 3396 patients with stroke and identified etiology were enrolled in this study (Fig. 1). Among them, 1483, 332, and 1581 were diagnosed with LAA, CE and SAO, respectively.

Patients had a median age of 62 years (IQR, 54.0–69.0) and 29.1% were female. Overall, 110 patients (3.2%) were identified as CHIP carriers with a variant allele frequency (VAF) ≥ 5%, which was almost consistent with previous reports in the general population [13]. Most variants occurred in five genes: *DNMT3A* (32 patients), *TET2* (18 patients), *GNAS* (6 patients), *JAK2* (6 patients) and *SETBP1* (5 patients). We rarely detected CHIP in patients under 40 years of age, and the prevalence of CHIP increased with age from 0.91% (4/439) in the age group of < 50 years, to 10.98% (18/164) in the age group of > 80 years.

### Association between CHIP and baseline characteristics

The baseline characteristics of the study population stratified by CHIP are presented in Table 1. As illustrated, the median age of CHIP carriers was approximately 6 years higher than that of non-CHIP carriers (*p* < 0.0001), and we did not observe significant differences in baseline characteristics between CHIP and non-CHIP carriers in stroke risk factors, except for current smoking status (*p* = 0.003). However, it was not statistically significant when adjusting for age and sex (OR 1.31, 95%CI 0.78–2.20, *p* = 0.31). Likewise, laboratory values did not differ between the two groups, except for hemoglobin level (*p* = 0.01). The median time from onset of symptom to hospital arrival for all participants was 1 day (IQR, 0–2).

**Table 1:** Baseline characteristics of the study population stratified by CHIP

Characteristic	Total (*n* = 3396)	Non-CHIP (*n* = 3286)	CHIP (*n* = 110)	*p* value
Age (y), median (IQR)	62.0 (54.0–69.0)	62.0 (54.0–69.0)	68.5 (64.0–76.0)	<0.001
Age, *n* (%)				<0.001
< 40	71 (2.1)	71 (2.2)	0	
40–49	368 (10.8)	364 (11.1)	4 (3.6)	
50–59	912 (26.9)	900 (27.4)	12 (10.9)	
60–69	1199 (35.3)	1157 (35.2)	42 (38.2)	
70–79	682 (20.1)	648 (19.7)	34 (30.9)	
≥ 80	164 (4.8)	146 (4.4)	18 (16.4)	
Female, *n* (%)	989 (29.1)	945 (28.8)	44 (40.0)	0.01
BMI (kg/m^2^), mean ± SD	24.9 ± 3.3	24.9 ± 3.3	24.7 ± 3.5	0.51
Current smoking, *n* (%)	1165 (34.3)	1142 (34.8)	23 (20.9)	0.003
Drinking, *n* (%)	594 (17.5)	582 (17.7)	12 (10.9)	0.06
Medical history, *n* (%)				
IS/TIA	743 (21.9)	717 (21.8)	26 (23.6)	0.65
CHD	334 (9.8)	322 (9.8)	12 (10.9)	0.70
Hypertension	2178 (64.1)	2102 (64.0)	76 (69.1)	0.27
Diabetes mellitus	841 (24.8)	817 (24.9)	24 (21.8)	0.47
Hypercholesterolemia	277 (8.2)	270 (8.2)	7 (6.4)	0.48
NIHSS				
Median (IQR)	3.0 (1.0–5.0)	3.0 (1.0–5.0)	3.0 (1.0–5.0)	0.80
TOAST				0.23
LAA	1483 (43.7)	1430 (43.5)	53 (48.2)	
CE	332 (9.8)	318 (9.7)	14 (12.7)	
SAO	1581 (46.6)	1538 (46.8)	43 (39.1)	
Laboratory index				
hsCRP	5.6 ± 18.8	5.6 ± 19.1	3.4 ± 5.7	0.53
HGB	142.2 ± 16.2	142.3 ± 16.2	138.9 ± 15.1	0.01
WBC	7.1 ± 2.1	7.1 ± 2.1	7.2 ± 2.3	0.70
MONO	0.4 ± 0.2	0.4 ± 0.2	0.4 ± 0.2	0.53
NEUT	4.7 ± 2.1	4.7 ± 1.9	5.3 ± 5.6	0.64
TG	1.6 ± 1.0	1.6 ± 1.0	1.5 ± 0.6	0.59
LDL	2.5 ± 1.0	2.4 ± 1.0	2.6 ± 1.1	0.21
HDL	1.0 ± 0.3	1.0 ± 0.3	1.0 ± 0.3	0.09
CHOL	4.1 ± 1.2	4.1 ± 1.2	4.2 ± 1.2	0.55
HCY	19.4 ± 12.9	19.4 ± 12.8	20.0 ± 15.5	0.98
Time from symptom onset to arrival at hospital, day(s)				
Median (IQR)	2.0 (1.0–4.0)	2.0 (1.0–4.0)	2.0 (1.0–4.0)	0.81

*BMI* body mass index, *CE* cardioembolism, *CHD* coronary heart disease, *CHIP* clonal hematopoiesis of indeterminate potential, *CHOL* total cholesterol, *HCY* homocysteine, *HDL* high density lipoprotein, *HGB* hemoglobin, *hsCRP* high-sensitivity C-reactive protein, *IQR* interquartile range, *IS* ischemic stroke, *LAA* large-artery atherosclerosis, *LDL* low-density lipoprotein, *MONO* monocyte count, *NEUT* Neutrophil count, *NIHSS* National Institutes of Health Stroke Scale score, *SAO* small-artery occlusion, *TIA* transient ischemic attack, *WBC* white blood cell count, *TG* triglyceride

### Association of CHIP with the risk of unfavorable functional outcome

**Fig. 2:**
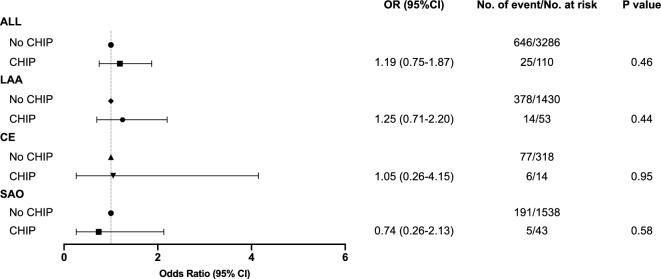
Unfavorable functional outcome stratified by CHIP carrier status in different etiology groups. A forest plot of the risk of unfavorable functional outcomes in subgroup analysis is shown. Odds ratios (ORs) were calculated using a logistic regression model and adjusted by stabilized inverse probability weights (IPTW). Variables for adjustments included age, sex, BMI, smoking, drinking, and history of disease (i.e., hypertension, diabetes mellitus, and hyperlipidemia). *CHIP* clonal hematopoiesis of indeterminate potential, *OR* odds ratio, *95% CI* 95% confidence interval, *LAA* large-artery atherosclerosis, *CE* cardioembolism, *SAO* small-artery occlusion

During the 3-month follow-up, 646 of 3286 non-CHIP-driver carriers (22.7%) and 25 of 110 CHIP carriers (19.7%) had unfavorable functional outcomes. Among the five most common genes, *JAK2* carriers had the highest incidence of unfavorable functional outcome (50%). Logistic regression analysis revealed that CHIP had no significant effect on the unfavorable functional outcome of all patients (OR 1.19, 95% CI 0.75–1.87, *p* = 0.46) (Fig. 2).

We further analyzed the association between CHIP and unfavorable functional outcomes under different inflammation statuses according to hsCRP levels in different etiology groups, and found that in patients diagnosed with LAA, 10 (58.8%) CHIP carriers and 182 (35.0%) non-CHIP carriers had unfavorable functional outcomes in patients with hyper-inflammation status. Logistic regression analysis showed that CHIP increased the risk of unfavorable functional outcomes among patients with higher hsCRP levels (OR 2.45, 95% CI 1.00–5.98, *p* = 0.049), but not in patients with hsCRP ≤ median (OR 0.30, 95%CI 0.08–1.13, *p* = 0.08; p_interaction_ = 0.01) (Table 2). Similar results were not observed in other etiology groups.

**Table 2:** Unfavorable functional outcome stratified by CHIP carrier status and hsCRP levels

	CHIP, *n* (%)	No CHIP, *n* (%)	Unadjusted OR (95%CI)	*p* value	^a^Adjusted OR (95%CI)	*p* value	*p*_interaction_
ALL							
hsCRP > median	18/52 (34.6)	409/1644 (24.9)	1.60 (0.89–2.86)	0.11	1.53 (0.83–2.80)	0.17	0.33
hsCRP ≤ median	7/58 (12.1)	237/1642 (14.4)	0.81 (0.37–1.81)	0.61	0.95 (0.46–1.98)	0.90	
LAA							
hsCRP > median	10/21 (47.6)	238/720 (33.1)	1.84 (0.89–2.86)	0.17	2.45 (1.00–5.98)	0.049	0.01
hsCRP ≤ median	4/32 (12.5)	140/710 (19.7)	0.58 (0.20–1.69)	0.32	0.30 (0.08–1.13)	0.08	
CE							
hsCRP > median	4/6 (66.7)	54/160 (33.8)	3.93 (0.70–22.12)	0.12	2.29 (0.28–19.02)	0.44	0.49
hsCRP ≤ median	2/8 (25.0)	23/158 (14.6)	1.96 (0.37–10.29)	0.43	0.78 (0.08–7.43)	0.83	
SAO							
hsCRP > median	3/24 (12.5)	113/765 (14.8)	0.82 (0.24–2.81)	0.76	0.62 (0.16–2.45)	0.49	0.71
hsCRP ≤ median	2/19 (10.5)	78/773 (10.1)	1.05 (0.24–4.62)	0.95	0.92 (0.18–4.73)	0.92	

*CE* cardioembolism, *CHIP* clonal hematopoiesis of indeterminate potential, *hsCRP* high-sensitivity C-reactive protein, *LAA* large-artery atherosclerosis, *OR* odds ratio, *95% CI* 95% confidence interval, *SAO* small-artery occlusion

^a^Odds ratios (ORs) were calculated using a logistic regression model and adjusted by stabilized inverse probability weights (IPTW) with age, gender, sex, BMI, smoking, drinking, and history of disease (i.e., hypertension, diabetes mellitus and hyperlipidemia)

### Genetic IL-6 signaling deficiency attenuates the risk of unfavorable functional outcome in patients with CHIP

Considering that inflammatory status might be the underlying mechanism between CHIP and unfavorable functional outcomes, we further investigated the potential efficacy of targeting a specific inflammatory pathway to improve unfavorable functional outcomes in patients with CHIP. Overall, 2230 (55.8%) patients had the *IL6R p.Asp358Ala* variant. Of these, 19.33% (431/2230) had unfavorable functional outcomes at 3 months. In 1166 patients without the *IL6R p.Asp358Ala* variant, 20.58% (240/1166) had unfavorable functional outcomes at 3 months. Logistic regression analysis showed that the presence of the *IL6R p.Asp358Ala* variant did not improve the functional outcome in all patients (OR 0.92, 95% CI 0.77–1.10, *p* = 0.36). Notably, the presence of the *IL6R p.Asp358Ala* variant significantly decreased the risk of unfavorable functional outcomes in CHIP carriers (OR 0.30, 95% CI 0.12–0.76, *p* = 0.01,) but not in non-CHIP carriers (OR 0.96, 95% CI 0.80–1.15, *p* = 0.68) (Table 3). The interaction between CHIP and the *IL6R p.Asp358Ala* variant was statistically significant (*p*_interaction_ = 0.02). Further analysis in different etiology groups showed that the effect of the *IL6R p.Asp358Ala* variant was significant only in patients with LAA (*p*_interaction_ = 0.001), but not in patients with CE and SAO.

**Table 3:** Unfavorable functional outcome stratified by CHIP carrier status and variant of *IL-6R*

	*IL6R p.Asp358Ala* *n* (%)	No *IL6R p.Asp358Ala* *n* (%)	Unadjusted OR (95%CI)	*p* value	^a^Adjusted OR (95%CI)	*p* value	*p*_interaction_
ALL							
CHIP	10/68 (14.7)	15/42 (35.7)	0.31 (0.12–0.78)	0.01	0.30 (0.12–0.76)	0.01	0.02
No CHIP	421/2162 (19.5)	225/1124 (20.0)	0.97 (0.81–1.16)	0.71	0.96 (0.80–1.15)	0.68	
LAA							
CHIP	3/32 (9.28)	11/21 (52.4)	0.09 (0.02–0.41)	0.002	0.10 (0.02–0.42)	0.002	0.001
No CHIP	253/903 (33.1)	125/500 (25.0)	1.12 (0.87–1.44)	0.37	1.10 (0.85–1.42)	0.46	
CE							
CHIP	4/10 (40.0)	2/4 (50.0)	0.67 (0.07–6.87)	0.73	0.66 (0.07–6.44)	0.72	0.83
No CHIP	51/221 (23.1)	26/97 (26.8)	0.82 (0.47–1.42)	0.48	0.85 (0.49–1.47)	0.56	
SAO							
CHIP	3/26 (11.5)	2/17 (11.8)	0.98 (0.15–6.57)	0.98	0.93 (0.14–6.23)	0.94	0.87
No CHIP	117/1011 (11.6)	74/527 (14.0)	0.80 (0.59–1.10)	0.16	0.79 (0.58–1.07)	0.13	

*CE* cardioembolism, *CHIP* clonal hematopoiesis of indeterminate potential, *LAA* large-artery atherosclerosis, *OR* odds ratio, *95% CI* 95% confidence interval, *SAO* small-artery occlusion

^a^Odds ratios (ORs) were calculated using a logistic regression model and adjusted by stabilized inverse probability weights (IPTW) with age, gender, sex, BMI, smoking, drinking, and history of disease (i.e., hypertension, diabetes mellitus and hyperlipidemia)

## Discussion

In this prospective cohort study of stroke patients, we found that the presence of CHIP was associated with the risk of short-term unfavorable functional outcomes in patients with LAA under hyper-inflammation, and that a genetic IL-6 signaling deficiency could attenuate the risk of unfavorable functional outcomes in patients with LAA stroke and CHIP.

Previous studies have shown that CHIP is associated with coronary heart disease by increasing the burden of atherosclerosis. The occurrence of CHIP increased the median coronary artery calcification scores of people without incident coronary heart disease by more than three times [2]. Atherosclerosis is one of the most common etiologies of stroke [14]. According to the TOAST classification [5], patients with clinical and brain imaging evidence of either significant stenosis (> 50%) or occlusion of a major brain artery or branch cortical artery were classified as having LAA. Inflammatory regulation is an important mechanism in the relationship between atherosclerosis and CHIP. Previous animal experiments have verified the causality between CHIP and atherosclerosis in *Ldlr−/−* mice. This causality was mainly shown by generating a pool of macrophages with elevated transcript levels of inflammatory markers, such as interleukin-6 (IL-6) [2]. Therefore, hsCRP levels were used for subgroup analysis to evaluate the association between CHIP and unfavorable functional outcome, whose production is stimulated by IL-6. We found that CHIP had different effect at different inflammation statuses only in patients with LAA stroke; it increased the risk of unfavorable functional outcomes only under higher levels of hsCRP. Accordingly, we speculated that the inflammation status of atherosclerosis might be a critical factor between CHIP and unfavorable functional outcomes, and the damage of CHIP to the brain may be aggravated when some inflammatory pathways are activated, which results in unfavorable short-term functional prognosis after having a stroke. However, more extensive experiments should be performed to provide a definitive causal relationship between CHIP and inflammatory and neurological functional outcomes.

Our data indicated that the IL-6 signaling blockade might be particularly beneficial to individuals with CHIP classified in LAA. IL-6 is the main driver of the production of the pro-inflammatory cytokine of CRP production in the liver, which is associated with an increased risk of ischemic stroke and is involved in the process of ischemic brain injury by binding to IL-6R to initiate intracellular signaling [15, 16]. IL-6 plays a comprehensive role in brain ischemic injury, as an inflammatory mediator in the acute phase and as a neurotrophic mediator during the subacute and prolonged phases. The potential of the application of IL-6 as a therapeutic agent for ischemic stroke has been a subject of interest [17]. Previous studies have shown that targeting IL-6 signaling pathways is beneficial to patients with atherosclerosis and has emerged as a key factor in managing atherothrombosis [18]. Ziltivekimab, a fully human monoclonal antibody directed against the IL-6 ligand, markedly reduced inflammatory biomarkers and thrombosis in atherosclerosis [3]. A recent study suggested that CHIP carriers with genetic deficiency of IL-6 signaling (by carrying IL6R p.Asp358Ala versus wild-type) had a greater reduction in cardiovascular disease risk when compared to non-CHIP carriers with genetic IL-6 signaling deficiency [4]. Our current study confirmed that individuals with *IL6R p.Asp358Ala* and CHIP had a lower risk of unfavorable functional outcome than those without CHIP in patients with LAA. We believe that blocking IL-6 signaling might be beneficial in patients with LAA and CHIP.

### Limitation

First, the average sequencing depth of WGS in this study was 30x, which might have slightly reduced the detection of CHIP. However, a previous study showed that even very low-depth WGS (1 × depth/4 × depth) could accurately assess the most common and low-frequency variations detected by high-depth whole-exome sequencing (75 × depth) [19]. Second, the sample size in some etiology groups was not adequately large, which might have restricted our analyses. Our results need to be confirmed in larger cohorts and tested in different ethnic populations around the world. Third, many previous studies have only focused on the most common driver of CHIP mutations to minimize potential heterogeneity that exists in CHIP driver mutation [4]; however, we included 74 genes in our study to fully evaluate the role of CHIP, which might have affected the results. Further experimental studies are required to identify the inflammatory mechanisms of CHIP in ischemic brain injury.

## Conclusion

In conclusion, our data revealed that CHIP is associated with an increased risk of unfavorable functional outcomes in patients with LAA stroke and hyper-inflammation. Genetic IL-6 signaling deficiency could attenuate the risk of unfavorable functional outcomes in CHIP carriers, especially in patients with LAA. Future studies are needed to validate the causality between CHIP, inflammation and unfavorable functional outcomes and discuss the impact of IL-6 pathway-targeted therapies to improve functional outcomes in CHIP carriers.

## Data Availability

The data supporting the findings of this study are available upon request from the corresponding author.
